# The Role of Toll and Nonnuclear NF-κB Signaling in the Response to Alcohol

**DOI:** 10.3390/cells12111508

**Published:** 2023-05-30

**Authors:** Nigel S. Atkinson

**Affiliations:** Department of Neuroscience and The Waggoner Center for Alcohol and Addiction Research, The University of Texas at Austin, Austin, TX 78712, USA; nigela@utexas.edu

**Keywords:** alcohol, addiction, innate immune system, neuroimmune, Drosophila, NF-κB, Toll

## Abstract

An understanding of neuroimmune signaling has become central to a description of how alcohol causes addiction and how it damages people with an AUD. It is well known that the neuroimmune system influences neural activity via changes in gene expression. This review discusses the roles played by CNS Toll-like receptor (TLR) signaling in the response to alcohol. Also discussed are observations in Drosophila that show how TLR signaling pathways can be co-opted by the nervous system and potentially shape behavior to a far greater extent and in ways different than generally recognized. For example, in Drosophila, TLRs substitute for neurotrophin receptors and an NF-κB at the end of a TLR pathway influences alcohol responsivity by acting non-genomically.

## 1. Introduction

In the United States, approximately 5% of individuals have an alcohol-use disorder (AUD) and, excluding COVID-19 infection, a third of preventable deaths are attributed to alcohol misuse [1,2]. Choices for treating individuals who have an AUD are very limited. Only three drugs were approved for this purpose. They are disulfiram, naltrexone, and acamprosate. Even with these, the success rate of treating alcohol-addicted individuals is dismal. During the first year of treatment, two-thirds of individuals have bouts of heavy drinking [3], while the best three-year average shows a ~25% rate of recidivism [4]. The rational treatment of alcohol-use disorders is dependent on understanding the mechanics of alcohol addiction. In this document, alcohol refers solely to ethanol.

### 1.1. Toll-like Receptors

Many consequences of alcohol misuse are linked to Toll-like receptor (TLR) pathways, which mediate alcohol-induced immune and neuroimmune activation that can lead to organ damage and to neurodegeneration (reviewed in [5]). Alcohol modulation of mammalian TLR pathways also appears to underlie behaviors associated with alcohol addiction.

In Drosophila, Toll receptor signaling was first shown to have a central role in dorsal-ventral pattern formation (see the review [6]) and was later shown to be used in adult flies as a major signaling pathway of the innate immune system—a role that was then shown to be conserved in mammals [7,8]. The name of the gene family, Toll-like receptors, was coined from the name of the *Drosophila melanogaster Toll* gene. The gene was identified in a genetic screen for mutations that interfere with dorsal-ventral patterning of the fly embryo [9]. The first alleles characterized were gain-of-function which cause ventralization of the embryo, while loss-of-function alleles cause dorsalization of the embryo. Ventralization refers to the developmental replacement of the dorsal for the ventral cell fate (dorsalization has the opposite meaning). Historically, Drosophila geneticists named genes as a reminder of their mutant phenotype and/or to amuse themselves. In this case, the German word toll translates to terrific, brilliant, stunning, smashing, and perhaps groovy. One of the discoverers, the Nobel Laureate Christiane Nüsslein-Volhard, explained that the name Toll has the intent of the English word “wow” [10].

The use of Toll signaling in dorsal-ventral pattern formation is not conserved in vertebrates [10]. However, Toll-like receptors or their orthologs play central roles in immune signaling in plants, invertebrates, and vertebrates, indicating that the ancestral role of Toll signaling is immunity. It appears that early in the evolutionary origin of the insect lineage, Toll signaling began to be co-opted for use in embryonic dorsal-ventral patterning. In holometabolous insects (such as Drosophila; holometabolous means that these animals undergo complete metamorphosis from larvae to pupa to adult), Toll is the major controller of dorsal-ventral patterning, while in hemimetabolous insects (these undergo incomplete metamorphosis in which the juvenile resembles the adult), Toll plays a minor patterning role. Exceptions to this simple dichotomy exist and have led to the proposal that convergent evolution has more than once selected Toll as the primary dorsal-ventral patterning receptor [9,11].

Humans have eleven TLR family members (TLR1-10 plus Cd180), rodents have thirteen (TLR1-9, 11-13 and CD180/RP105, in rodents TLR10 is a pseudogene), while Drosophila have nine [12,13,14]. Mammalian TLR orthologs are numbered concordantly with each other. Drosophila TLR numbering is not concordant with mammalian numbering. CD180/RP105 expression parallels TLR4 in antigen-presenting cells, the protein associates with other TLRs, and phylogenetically, the gene belongs to the TLR4 subfamily [15,16].

TLRs are usually divided into cell surface receptors and intracellular endosomal receptors. Most of our understanding of the subcellular localization of TLRs comes from the analysis of immune cells. In immune cells, it was considered dogma that the cell membrane Toll-like receptors are TLR1, 2, 4, 5, and 6 and the endosomal Toll-like receptors are TLR3, 7, 8, 9, and in mice, 11, 12, and 13. However, recent evidence indicates that this segregation is not absolute. TLR4 was shown to also signal from the endosome [17,18] and furthermore, in some cell types, TLR3, 7, and 9 were found on the cell surface. Only TLR8 was not yet reported to also reside in the cell membrane. It is possible that the non-canonical localization of the endosomal TLRs reflects an error in receptor transport. However, at least for TLR7, this supposition is undermined by the observation that functionally important TLR7 shows cell-type specific localization to endosomes in cortical and hippocampal neurons and to the cell membrane in at least some sensory neurons [17,19,20,21].

TLR1, 2, and 6 respond to diacylated and/or triacylated lipopeptides, TLR4 responds to bacterial lipopolysaccharides, and TLR5 responds to flagellin. TLR3, 7, 8, 9, and 13 respond to non-self nucleic acids, while TLR11 responds to microbial protein antigens and TLR11 and TLR12 respond to parasitic protein antigens [13,17,18,22].

Intracellular adaptor proteins that associate with TLRs determine which transcription factors are activated when the receptor is stimulated. TLR1, TLR2, TLR4, TLR5, TLR6, TLR7, TLR8, and TLR9 signal through the MyD88 adapter, while TLR3 and intracellular TLR4 signal through the TRIF adapter. MYD88 signaling is best known for its activation of NFKBs but can also cause AP1 and CREB activation. TRIF signaling leads to the activation of an IRF-type transcription factor (IRF3, 5, and/or 7) and, in a delayed fashion, TRIF signaling can also activate NFKB transcription factors [23] (see the excellent figures in Gay et al. [17] that summarize TLR localization and TLR signaling pathways). In the brain, alcohol directly or indirectly activates TLRs and induces the expression of all or almost all of the TLRs [24,25]. This promotes an escalating proinflammatory state.

### 1.2. TLRs and Alcohol Responses

Among the human TLRs, TLR4 is the most highly studied and was the first TLR linked to important alcohol responses. Alcohol activates TLR4 signaling in astroglia and microglia, leading to neuroinflammation and, probably, alcohol-induced neurodegeneration [26]. These responses are correlated with increased anxiety and an increase in cognitive defects. The suppression of TLR4 activity protects against these consequences [27]. Some drugs that modulate alcohol consumption and alcohol impairment were shown to modulate TLR4. For instance, isomers of naltrexone and naloxone that are inactive on opioid receptors but retain their capacity to antagonize TLR4 decrease alcohol consumption or alcohol impairment (summarized in [28], but see below).

The neuronal GABA_A_R α2 subunit was shown to physically associate with and activate TLR4. Alcohol-preferring P rats have both increased GABA_A_ α2 and TLR4 receptor expression in the central amygdala and ventral tegmental area (VTA). Activation of GABA_A_R α2-associated TLR4 was shown to stimulate the CREB transcription factor. At least in the VTA, CREB activation promotes increased corticotropin-releasing factor (CRF) and tyrosine hydroxylase gene expression. In a feedback loop, activation of CRF can further stimulate TLR4. The GABA_A_R α2/TLR4 signaling combination was reported to promote binge alcohol drinking in rats and RNAi experiments showed that knockdown of either GABA_A_R α2 or TLR4 reduces binge drinking [29,30]. These studies appeared to tightly link TLR4 activation to the promotion of alcohol consumption in a way that is pharmacologically attractive.

However, not all studies showed a reliable connection between alcohol drinking and TLR4 (itemized in [31]). A report by the Integrative Neuroscience Initiative on Alcoholism tried to resolve the issue of whether or not TLR4 was mechanistically involved in the development of uncontrolled drinking. Using mice and rats and multiple alcohol drinking assays, this group appeared to have resolved this issue, leading to the conclusion that TLR4 does not directly modulate drinking itself, but does consistently affect the acute sedative effects of alcohol and the kinetics of GABA_A_R receptors [31].

However, TLR4 clearly has a role in alcohol-induced proinflammatory responses and the cognitive problems associated with inflammation [5,26,27]. In addition, in human studies, the methylation status of a TLR4 promoter-associated CpG and the level of TLR4 gene expression correlate with symptoms of depression [32]. Perhaps the relationship to depression represents an indirect path for TLR4 to influence alcohol consumption.

Although TLR4 by itself does not directly affect alcohol consumption, other TLR receptors appear to. With regard to the propensity to drink alcohol, the genetic ablation of the TLR2 gene in mice decreases alcohol drinking in both the continuous access two-bottle choice paradigm, and the intermittent access drinking-in-the-dark paradigm [33] (in addition, a TLR2 null mutation also almost completely eliminates the sensitivity to alcohol sedation [34]). Intriguingly, alcohol promotes a physical interaction between TLR2 and TLR4 in microglia [35]. Perhaps the association with TLR2 contributes to the sometimes-observed link between TLR4 and alcohol consumption. In mice, components of the TLR3/TRIF signaling pathway are induced by voluntary alcohol consumption [36]. In addition, mouse TLR3 activation modulates alcohol consumption in a sexually dimorphic manner [37,38,39] and in rats, TLR3 activation promotes alcohol self administration in both male and female animals [40]. Finally, in mice, the TLR7 agonist R484 promotes alcohol drinking [41] and in rats, a different TLR7 agonist was demonstrated to increase alcohol consumption [41,42]. Although not restricted to these TLRs, in human alcoholic brains, the levels of TLR2, TLR3, TLR7, and HMGB1 (a TLR ligand) correlate with lifetime alcohol consumption [41,43].

### 1.3. TLRs as Neurotrophin Receptors

Unlike mammalian TLRs, the Drosophila Toll receptor does not function directly as a pattern recognition molecule. Instead, in the fat body, it was shown that a collection of secreted pattern recognition molecules bind pathogen components and trigger the proteolytic cleavage of the Spz cytokine. This activated Spz isoform binds the Toll receptor, promoting its dimerization and signaling. In the fat body, this causes the expression of antimicrobial peptides [44]. However, it is unknown whether infection also activates neuronally expressed Toll-like receptors.

There is substantial evidence for neurotrophic interactions in the Drosophila CNS despite the fact that Drosophila lack obvious orthologs of mammalian Trk, p75NTR, or Sortilin neurotrophin receptor genes [45,46]. In addition to its role as a cytokine in the fat bodies, in the nervous system, *spz* gene family members function as neurotrophic factors, and some or all fly TLRs act as neurotrophic receptors important for neuronal survival and death [47]. While the study of the role of neurotrophic factors and receptors was unevenly studied in flies, it was clear that Spz, DNT1 (aka Spz-2), Spz5 (aka DNT2), and Spz3 function as neurotrophic factors and that Toll, Toll-2, Toll-6, Toll-7, and Toll-8 serve as neurotrophic receptors [46,47,48,49,50,51]. The Toll receptor was shown to be activated by Spz, Spz2, and Spz5; Toll-6 by Spz5; Toll-7 by Spz1, Spz2, and Spz5; while Spz3 was shown to signal through Toll-8 [46,49,52]. The fly brain showed a topologically distinct expression of TLRs, suggesting that TLRs could provide targeted neurotrophic activity that organizes the brain into distinct modules [53]. It is not known whether, in flies, the neurotrophic activity of TLRs involve signaling through the Dif NF-κB.

In mammals, neurotrophins are thought to play important roles in neuronal development, in pre- and post-synaptic mechanisms of synaptic plasticity, and in learning and memory. Neurotrophins were identified as important targets for treatment of psychiatric disorders [54]. Furthermore, in mammals, neurotrophic factors are linked to alcohol consumption in meaningful ways. Alcohol enhances the production of brain-derived neurotrophic factor (BDNF) in the dorsal striatum, and manipulating BDNF expression alters alcohol consumption [55].

It is not yet clear whether, as in flies, mammalian TLRs should be considered neurotrophin receptors. However, there is evidence that mammalian TLRs modulate adult CNS neurogenesis and dendritic arborization [53,56]. Specifically, TLR2, and TLR5 promote neuronal stem cell (NSC) differentiation, while TLR3 and TLR4 signaling suppresses NSC differentiation and proliferation. Furthermore, TLR3 and TLR7 act as negative regulators of axonal growth and, in addition, dendritic spine density is stimulated by TLR3 and TLR8 activity (reviewed in [57]). Furthermore, mammalian neurons make use of TLRs to alter the neuronal response to stimuli. Mammalian sensory neurons have co-opted TLRs for sensing danger signals. In nociceptive neurons, TLRs2, 3, 4, and 7 functionally couple with TRPV1 and/or TRPA1 channels in the production of itch and pain responses. In addition, flagellin activation of TLR5 in dorsal spinal cord neurons induces the sensation of mechanical itch and scratching in mice (reviewed in [58]).

### 1.4. Synaptically Localized NF-κBs

Clearly, signaling through more than one TLR pathway is relevant for the pharmacological manipulation of alcohol consumption. Fortunately, all mammalian TLRs can activate NF-κBs, making this transcription factor an integration point for most TLR signaling events [17]. Because of the role of NF-κBs in inflammation, a great many small molecule NF-κB inhibitors and activators were developed. In mouse models, drugs that inhibit NF-κB activation, such as sulfasalazine, amlexanox, and TPCA-1, suppress alcohol drinking and alcohol preference (reviewed in [28]).

In mammals, neuronally expressed NF-κBs were shown to be important for normal physiology of both excitatory and inhibitory neurons. They were shown to influence synaptic plasticity, including long-term potentiation, long-term depression, and synaptic structure and the capacity for animal learning, memory, and cognition. Furthermore, NF-κBs are found in the synaptic compartment and post-synaptically, where they can be activated by Ca^2+^ influx though NMDA receptors and L-type Ca^2+^ channels [59,60]. In general, it was shown or it was assumed that the activation of synaptic NF-κBs causes them to translocate to the nucleus where they produce their effects by modulating gene expression (reviewed in [60,61]). Many consequences of alcohol misuse are linked to TLR-mediated activation of NF-κB transcription factors [62]. Furthermore, in a human genome–wide association study, eight SNPs across the *NFKB1* gene showed significant association with alcohol dependence [63] and in mice, alcohol consumption was reduced by I-κB kinase inhibition (an activator of NF-κB activity; [64]). Non-immune roles of NFKBs in the nervous system and in relation to drug addiction was reviewed in 2017 by Nennig and Schank [65].

**Figure 1:**
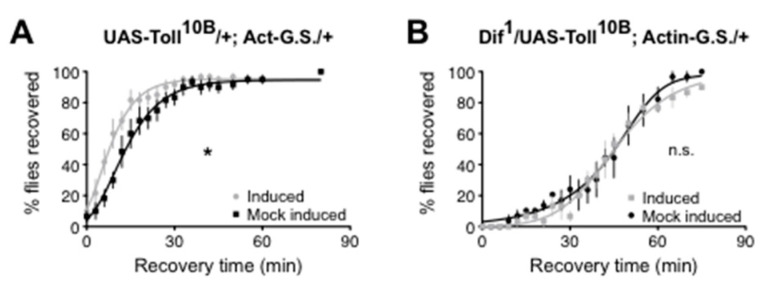
Reduced Dif activity epistatically masks the effect of a constitutively active *Toll* allele. Recovery from alcohol sedation shown for a population of flies. (**A**) Induction of a constitutively active *Toll* allele (UAS-Toll10B), using gene switch technology (Act-G.S.), causes a population of flies to recover more quickly from alcohol sedation. * *p* = 0.03. (**B**) A heterozyogous loss-of-function *Dif* ^1^ allele suppresses the alcohol resistance phenotype produced by a *Toll* gain-of-function allele. n.s. = not significant. Published in [66].

Troutwine et al. [66] demonstrated that, as for mammalian TLR4, the Drosophila Toll signaling pathway affects alcohol sensitivity. Each step in the canonical Toll to NF-κB pathway was tested using mutation, RNAi, and/or transgenes. Activation of the pathway produced resistance to alcohol sedation, while inhibition of the pathway increased sensitivity to alcohol sedation. In adult flies, the NF-κB at the end of the Toll signaling pathway was shown to be encoded by the *Dif* gene. Confirmation that Toll signaling through the Dif NF-κB was relevant for the change in alcohol sensitivity was demonstrated in an epistasis experiment in which alcohol resistance produced by a *Toll* gain-of-function allele could be suppressed by a heterozygous *Dif* loss-of-function allele (Figure 1).

At times, a change in neural NF-κB activity within a brain region may be expected but not observed. It is understandable that focus could then shift to a brain region that shows the expected change in NF-κB activity (e.g., [67]). Such a change in focus may be warranted, but it might also be a result of the limitation of the assays used to detect activated NF-κB. Almost all experimenters define activated NF-κB as NF-κB that has translocated to the nucleus. Usually, nuclear extracts are prepared, incubated in vitro with DNA oligomers that specify consensus NF-κB-binding sites, and assayed by an ELISA-based protocol. However, this approach is dependent on the axiomatic assumption that all NF-κB effects are mediated by action in the nucleus. Were an important action of an NF-κB to not involve nuclear entry, its activation would be undetectable using this assay. In such a case, the switch to the brain region where an activated NF-κB acts in the nucleus could be a red herring. Data from flies suggest that some NF-κB-type proteins do not have to enter the nucleus and do not have to act as transcription factors in order to fundamentally affect animal behavior.

**Figure 2:**
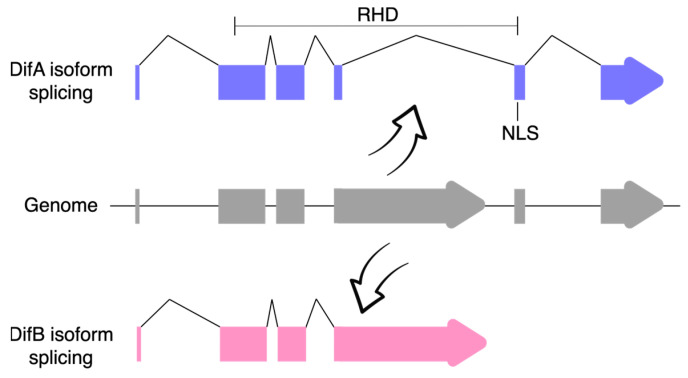
The *Dif* gene produces two protein isoforms by alternative mRNA processing. Note that DifB is missing part of the Rel-homology domain (RHD) and also the nuclear localization signal (NLS; domains as specified in Uniprot). UTRs are not shown.

Closer examination of the *Dif* gene showed that it produced two classes of mRNA via alternative mRNA splicing (Figure 2). In the DifA variant, the C-terminal exons encode 308 amino acids that include a nuclear localization signal (NLS) and a transactivation domain, whereas in the DifB variant, these exons were replaced by a single large exon that encodes 627 amino acids but does not recognizably include either an NLS or the transcriptional activation domain [68]. No obvious conservation of the B-specific exon was discovered in mammals.

A fly paralog of *Dif* is the *dorsal* gene. The *dorsal* and *Dif* genes are about 4 kb apart on chromosome 2—the obvious product of a tandem duplication. *Dif* and *dorsal* are highly similar and while they can at least partially substitute for one another when ectopically expressed, they do not do so under normal conditions, because they are expressed in different cells. The similarity between *dorsal* and *Dif* extends to their patterns of mRNA splicing. The *dorsal* gene also expresses two protein variants, one of which is Dorsal-A, which is analogous to DifA, and the other of which is Dorsal-B, which is analogous to DifB. A synopsis of what is known about Dorsal-B follows, and it is relevant to our understanding of DifB. The Dorsal-B and DifB variants show greater than 40% amino acid identity.

NF-κBs are prevented from entering the nucleus by an I-κB (Inhibitor of NF-κB). In flies, the relevant I-κB is encoded by the *cactus* gene. Two groups showed that, in larval body-wall muscle, the *dorsal*-encoded NF-κB could not be made to enter the nucleus even after suppression of Cactus/I-κB activity [68,69]. Instead, in larval muscle, Dorsal protein colocalized with the post-synaptic Dlg1 protein in the subsynaptic reticulum at type I postsynaptic boutons of the neuromuscular junction (NMJ). These boutons generate glutamatergic excitatory junction potentials. It was shown that, at the larval NMJ, Dorsal protein forms a halo around post-synaptic GluRIIA receptors and the suppression of Dorsal expression reduces GlurIIA abundance and synaptic efficacy. Furthermore, in larval muscle, an essentially identical effect on GlurIIA abundance was observed when two other Toll signaling pathway members (Cactus/I-κB and Pelle/IRAK) were depleted [68,69,70]. The effects of Dorsal on GluR synaptic levels were rigorously demonstrated to be post-transcriptional and consistent with modulation of receptor insertion at the synapse [69].

At the time that the original NMJ studies were conducted, the researchers did not know that *dorsal* expressed two protein isoforms. However, in 2015, Zhou et al. [68] showed that the Dorsal protein at the NMJ was the Dorsal-B variant. Furthermore, the Dorsal-B variant differs from the A variant in that Cactus is not responsible for Dorsal-B localization. Instead, Dorsal-B is required for the synaptic localization of Cactus. The strong similarity between Dorsal-B and DifB leads one to suspect that DifB behaves similarly to Dorsal-B, albeit in different cells.

In the study of Wijesekera et al. [71], it was shown that DifA is expressed in fat bodies. The fat body is a multifunctional organ that regulates carbohydrate and lipid metabolism and body size and is a major organ of the innate immune system [72]. Infection with fungi, Gram-positive bacteria, or even sterile wounding, activates the Toll signaling pathway in the fat body to trigger the secretion of a variety of antimicrobial peptides [73]. In the adult fat body, the NF-κB at the end of Toll signaling pathway is DifA.

Immunohistochemical staining shows that DifB is not expressed in fat bodies but, instead, is expressed in the brain where it is abundant in the mushroom bodies and moderately abundant in the antennal lobes and ventral nerve cord. Whereas DifA is not obviously expressed in the adult CNS. The mushroom bodies play a central role in learning and memory [74], and in flies, the mushroom bodies and pathways associated with learning and memory are frequently associated with alcohol-induced responses [75,76,77,78,79,80].

**Figure 3:**
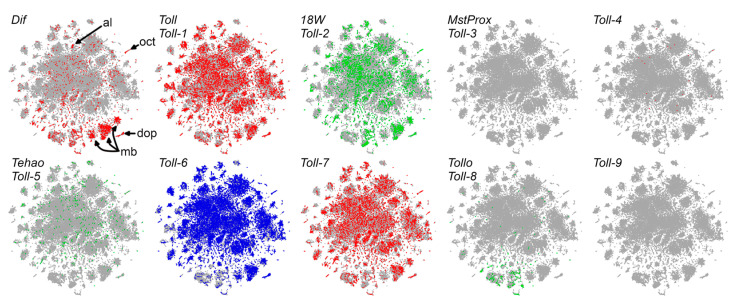
SCope display of single-cell sequencing data from Davie et al. [81] shows that *Dif* is expressed in mushroom bodies (mb = Kenyon cell of mushroom bodies) and antennal lobes (al) as confirmed by Wijesekera et al. using immunohistochemical staining [71]. Additionally seen is *Dif* expression in dopaminergic (dop) and octopaminergic-tyraminergic (oct) neurons. *Toll*, *18w* (Toll-2), Toll-6, and Toll-7 show expression that overlaps *Dif* in all of these areas according to the data from Davie et al. When appropriate, receptors are named with the conventional Drosophila name and a common numbered synonym. In this plot, the labeling of brain regions was by Davie et al. The data being displayed from the SCope archive are identified as Aerts_Fly_AdultBrain_filtered_57k. As described by Davie et al., in this dataset, there are 56,902 high-quality cells from 26 runs which were stringently filtered. Expression was visualized with the SCope viewer using the default settings provided by the authors, they are: SCENIC 25PC, 60 perplexity, Log transform, with Expression-based plotting. However, after gene selection, the SCope expression level sliders were set to display all expression levels with the same color intensity, so that the plots show where genes are expressed but not the relative level of expression in each cell type because we cannot sensibly assign meaning to different expression levels at this time.

SCope analysis of single-cell sequencing data [81] confirmed these immunohistochemical observations, showing *Dif* expression in the Kenyon cell neurons of the mushroom bodies and in the antennal lobes (Figure 3). In single-cell sequencing, Kenyon cell neurons can be identified by the strong expression of the *ey*, *Imp*, and *sNPF* genes. Strikingly, *Dif* is at least as good a Kenyon cell identifier as are these recognized marker genes.

Immunohistochemical staining can provide insight into the subcellular localization of proteins in the insect CNS. Fly central nervous system neurons are organized into neuropil comprised of synapse-rich axonal-dendritic projections surrounded by a rind of neuronal cell bodies [82,83]. Thus, in the insect brain, one can readily distinguish nuclear localization from synaptic localization. DifB immunoreactivity was observed to be highly localized to the synapse-rich neuropil away from the cell bodies. DifB was never observed within neuronal nuclei in adults or in larvae. This observation was then validated using biochemical subcellular fractionation which showed that DifB copurified in the synaptoneurosome fraction and away from the nuclear fraction [71]. This non-nuclear localization was observed even when the Toll signaling pathway was activated by infection [68,71].

**Figure 4:**
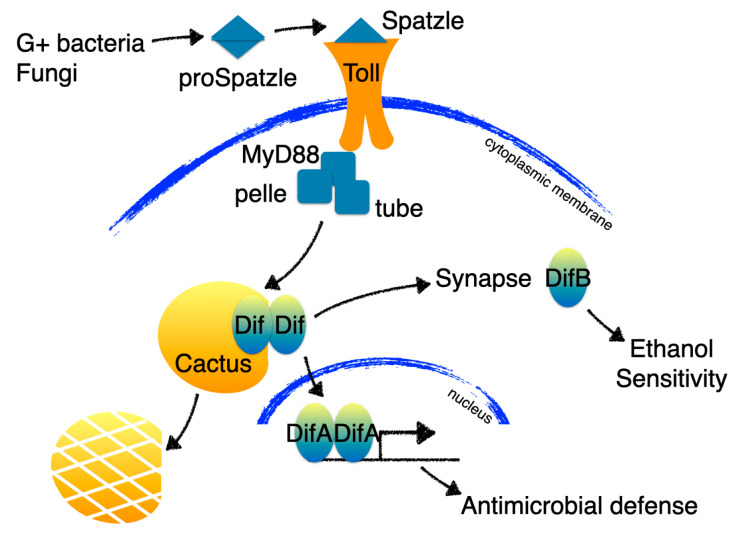
In the Drosophila fat body, Toll activation by infection (or through an unknown mechanism by alcohol) signals through the DifA NF-κB isoform to activate antimicrobial defenses. The CNS does not express the DifA nuclear-acting isoform but instead expresses a non-nuclear NF-κB isoform called DifB that is enriched in the synaptic compartment. Mutations in DifA affect the immune response but not alcohol sensitivity while DifB mutations have a complementary phenotype —affecting alcohol sensitivity but not immunity.

The non-overlapping expression of DifA and DifB results in distinct functional consequences when DifA expression is suppressed and when DifB expression is suppressed. A mutation that specifically eliminates DifA causes severe immunosuppression, but does not affect alcohol sensitivity. Conversely, a DifB-specific mutation does not seem important for immunity but results in increased alcohol sensitivity. Flies have compartmentalized distinct functions of Dif into different alternative splice variant isoforms (Figure 4; [71]).

### 1.5. Overlap of DifB and TLR Expression in the CNS

We were interested in identifying which Toll-like receptors might signal through Dif in the brain. Figure 3 shows that single-cell sequencing data indicate that four of the nine fly Toll-like receptors are coexpressed with DifB in both mushroom body and antennal lobe neurons. Toll, Toll-2, Toll-6, and Toll-7 are co-expressed with Dif in both Kenyon cells of the mushroom bodies and in the antennal lobes (Figure 3). While some fly Toll-like receptors can signal through transcription factors other than NF-κB, the Toll, Toll-2, Toll-6, and Toll-7 receptors are all known to use the canonical MyD88 signaling pathway that terminates with NF-κB activation [48,51]. Toll was already shown to affect alcohol sensitivity by signaling through the *Dif* NF-κB [66]. It is yet to be determined whether these additional Toll-like receptors do so as well.

## 2. Closing

Components of the Drosophila TLR signaling pathways act in ways that are not yet observed in mammals. Clearly, in the fly CNS, the neuroimmune system or at least components of the neuroimmune system were co-opted to directly modulate behavior via signaling through a TLR pathway. The non-nuclear NF-κB encoded by *Dif* is at a good position for implementing this TLR function. There is evidence that mammalian NF-κBs are also well-positioned to directly modulate synaptic activity. Some mammalian NF-κBs also localize to the synapse, and in response to Ca^2+^, some transition into the membrane—a localization at odds with a nuclear function [60]. Furthermore, Xie et al. [84] showed that in DRG neurons, a membrane-bound NF-κB non-transcriptionally slows the inactivation of voltage-gated sodium channels and speeds recovery from inactivation.

Because of their importance in immunity, TLR signaling molecules and NF-κB transcription factors are some of the most highly studied proteins in mammals. From these studies, the range of functions performed by these entities is well known and led to assumptions concerning how they act. Despite this, studying the proteins in an animal distantly related to any mammal highlights new capabilities of these molecules and new ways that neurons can co-opt immune signaling pathways for a different purpose. The demonstrated potential for novel use of innate signaling molecules by an insect CNS should lead one to suspect that the CNS of other animals might also find such uses beneficial. The fact that they were not reported to occur in Mammalia may arise from preconceptions concerning what these proteins can do. Certainly, the outsized role played by neuroimmune signaling in modulating behaviors associated with alcohol-use disorders was originally surprising. Perhaps work from Drosophila is indicating additional surprises await in the relationship between alcohol responses and how neuroimmune signaling modulates neural activity.
